# Left ventricular noncompaction as diagnosed by established cardiac magnetic resonance imaging criteria is not associated with increased adverse events compared to non-ischemic dilated cardiomyopathy

**DOI:** 10.1186/1532-429X-17-S1-P318

**Published:** 2015-02-03

**Authors:** Shermeen B Memon, Lara Bakhos, Nathan Bibliowicz, Rajeev R Fernando, Mark Rabbat, Thriveni Sanagala, Ari Goldberg, Mushabbar A Syed

**Affiliations:** 1 Cardiology, Loyola University, Chicago, IL, USA; 2 Radiology, Loyola University, Chicago, IL, USA

## Background

Left ventricular noncompaction (LVNC) is classified by the American Heart Association as a primary genetic cardiomyopathy and is attributed to defects in cardiac embryogenesis resulting in the intrauterine arrest of the compaction of the loose meshwork that makes up the fetal myocardium. From echocardiographic data, the prevalence of LVNC has been estimated at 0.05% of the general population. With the increasing use of cardiac magnetic resonance imaging (CMR), there has been a surge in the reports of patients with LVNC. Interestingly, many patients that have been diagnosed with non-ischemic dilated cardiomyopathy (NIDCM) have also been noted to have prominent left ventricular trabeculations. We sought to evaluate the difference in clinical outcomes in patients with NIDCM compared to those with LVNC as diagnosed by established CMR criteria.

## Methods

A retrospective analysis was performed on 71 patients diagnosed with NIDCM at a single tertiary care center who had undergone a CMR between January 1, 2012 and August 30, 2014. The diagnosis of cardiomyopathy was established based on clinical suspicion and a dilated left ventricle (LV) when indexed to body surface area. Baseline characteristics and clinical outcomes were obtained. Volumetric quantification was performed to obtain chamber volumes and ejection fractions (EF). The ratio of compacted:non-compacted myocardium was measured at end-diastole in both the 4- and 2-chamber orientations. The data was analyzed using analysis of variance (ANOVA) and Pearson's chi squared testing with SPSS Statistics for Windows.

## Results

Of 71 patients, 25% were found to meet the criteria of LVNC based on established CMR criteria. The mean age of individuals diagnosed with LVNC was 50.9 years as compared to 50.4 years (p=0.907). The incidence of prior stroke, diabetes mellitus, hypertension, end-stage renal disease, and cancer treated with chemotherapy or radiation did not differ between the two groups. The mean LVEF in both groups was 36% (p=0.992). There was no statistical difference in the mean number of heart failure admissions when comparing patients with LVNC and NIDCM (0.83 vs. 0.73, p=0.747). Both groups exhibited similar occurrences of ventricular (p=0.473) and atrial arrhythmias (p=0.204). For all patients, the most commonly trabeculated areas were the anterior and lateral walls, while the least was the septum. The apical segments were noted to have the most prominent trabeculations.

## Conclusions

This represents the largest study comparing the clinical outcomes of those patients with MRI defined LVNC to those with NIDCM. Our results demonstrate LVNC may not be prognostically different than NIDCM, suggesting that LVNC may be a morphological variant of NIDCM or perhaps that the current CMR criteria for LVNC need to be revised. Larger studies are necessary to better evaluate and understand LVNC.

## Funding

None.

**Table 1:** Patient Comparison of LVNC and NIDCM

	Individuals with ≥ 3 segments with non-compacted:compacted ratio ≥ 2.3n = 18 (%)	Individuals with < 3 segments with non-compacted:compacted ratio < 2.3 n = 53 (%)	p-value
**Baseline Characteristics:**			

Males	7 (38.9)	32 (60.4)	

Mean Age in Years	50.9 +/- 17.9	50.4 +/- 15.3	0.907

History of CVA	1 (5.6)	1 (1.9)	0.416

History of Diabetes Mellitus	4 (22.2)	16 (30.2)	0.516

History of Hypertension	9 (50)	27 (50.9)	0.945

History of ESRD	0 (0)	1 (1.9)	0.557

History of Cancer with Exposure to Chemotherapy/Radiation	3 (16.7)	7 (13.2)	0.715

**Outcomes:**			

Average Number of Heart Failure Admissions per Patient	0.83 +/- 1.0	0.73 +/- 1.2	0.747

Thromboembolic Events	2 (11)	3 (5.7)	0.435

SVT	6 (33.3)	10 (18.9)	0.204

NSVT or VT	2 (11.1)	11 (20.8)	0.473

Underwent ICD Placement	6 (33.3)	10 (18.9)	0.393

Underwent LVAD placement	1 (5.6)	0 (0)	0.084

Underwent Heart Transplantation	0 (0)	0 (0)	--

Mortality	0 (0)	1 (1.9)	0.557

**CMR features:**			

LV EF (%)	36.0 +/- 17.9	36.1 +/- 12.7	0.992

LV End Diastolic Volume Index (mL/m^2^)	139.0 +/- 49.0	122.0 +/- 41.4	0.157

LV End Systolic Volume Index (mL/m^2^)	93.8 +/- 53.6	81.9 +/- 41.6	0.333

LV Mass Index (g/m^2^)	58.9 +/- 19.5	65.0 +/- 20.8	0.277

RV EF (%)	45.4 +/- 12.4	46.1 +/- 10.3	0.816

RV End Diastolic Volume Index (mL/m^2^)	88.3 +/- 24.3	84.8 +/- 27.7	0.634

RV End Systolic Volume Index (mL/m^2^)	47.6 +/- 22.6	47.1 +/- 22.5	0.927

LA Volume Index (mL/m^2^)	64.6 +/- 18.7	54.8 +/- 23.9	0.122

Data displayed as n (%) or mean +/- standard deviation. CVA = cerebrovascular accident. ESRD = end-stage renal disease. SVT = supraventricular tachycardia. NSVT = non-sustained ventricular tachycardia. VT = ventricular tachycardia. ICD = implantable cardioverter defibrillator. LVAD = left ventricular assist device. CMR= cardiac magnetic resonance imaging. LV = left ventricle. EF = ejection fraction. RV = right ventricle. LA = left atrium. Means compared with ANOVA. Baseline characteristics and outcomes compared with Pearson's chi squared testing.

